# Implications of differences in expression of sarcosine metabolism-related proteins according to the molecular subtype of breast cancer

**DOI:** 10.1186/1479-5876-12-149

**Published:** 2014-05-28

**Authors:** Ja Kyung Yoon, Do Hee Kim, Ja Seung Koo

**Affiliations:** 1 Department of Pathology, Yonsei University College of Medicine, Severance Hospital, 50 Yonsei-ro, Seodaemun-gu, Seoul, South Korea

**Keywords:** Breast cancer, Metabolism, Molecular subtype, Sarcosine

## Abstract

**Background:**

The goal of this study was to investigate the expression of sarcosine metabolism-related proteins, namely glycine *N*-methyltransferase (GNMT), sarcosine dehydrogenase (SARDH), and l-pipecolic acid oxidase (PIPOX), in the different breast cancer subtypes and to assess the implications of differences in expression pattern according to subtype.

**Methods:**

We analyzed the expression of GNMT, SARDH, and PIPOX in a tissue microarray of 721 breast cancer cases using immunohistochemistry (IHC). We classified breast cancer cases into subtype luminal A, luminal B, HER-2, and triple negative breast cancer (TNBC) according to the status for the estrogen receptor (ER), progesterone receptor (PR), HER-2, and Ki-67. Sarcosine metabolism phenotype was stratified according to IHC results for GNMT, SARDH, and PIPOX: GNMT(+), SARDH and PIPOX(-) was classified as high sarcosine type; GNMT(-), SARDH or PIPOX(-) as low sarcosine type; GNMT(+), SARDH or PIPOX(+) as intermediate sarcosine type, and GNMT(-), SARDH and PIPOX(-) as null type.

**Results:**

Expression of sarcosine metabolism-related proteins differed significantly according to breast cancer subtype (GNMT, p = 0.005; SARDH, p = 0.012; tumoral PIPOX, p = 0.008; stromal PIPOX, p < 0.001). These proteins were the most frequently expressed in HER-2 type tumors and the least in TNBC. Sarcosine metabolism phenotype also varied according to breast cancer subtype, with high sarcosine type the most common in HER-2, and null type the most common in TNBC (p = 0.003). Univariate analysis revealed that GNMT expression (p = 0.042), tumoral PIPOX negativity (p = 0.039), and high sarcosine type (p = 0.021) were associated with shorter disease-free survival (DFS). Multivariate analysis also revealed GNMT expression was an independent factor for shorter DFS (hazard ratio: 2.408, 95% CI: 1.154-5.024, p = 0.019).

**Conclusion:**

Expressions of sarcosine metabolism-related proteins varied according to subtype of breast cancer, with HER-2 type tumors showing elevated expression of these proteins, and TNBC subtype showing decreased expression of these proteins. Expression of sarcosine metabolism-related proteins was also associated with breast cancer prognosis.

## Background

Sarcosine (N-methylglycine), a non-proteogenic amino acid produced in the synthesis and degradation of glycine, is produced by methyl group transfer from *S*-adenosylmethionine (SAM) to glycine by glycine *N*-methyltransferase (GNMT). Other major sarcosine-metabolizing enzymes - sarcosine dehydrogenase (SARDH) and I-pipecloioc acid oxidase (PIPOX) - detach the methyl group from sarcosine via oxidative demethylation to form glycine [1]. The role of sarcosine extends beyond its identity as a non-proteogenic amino acid; it is also a potential oncometabolite. Prostate cancer studies have reported that sarcosine is a sensitive tumor biomarker and suggest its involvement in tumor progression and metastatic processes [2-4].

Breast cancer is clinically, histopathologically, and molecularly heterogeneous. Efforts to classify tumors with similar characteristics have resulted in subtyping of breast cancer into luminal A, luminal B, HER-2, normal breast-like, and basal-like types through genetic profiling analysis [5,6]. A separate set of subtyping criteria relies on the expression of important therapeutic markers: estrogen receptor (ER), progesterone receptor (PR), and HER-2, from which the term triple negative breast cancer (TNBC) is derived. TNBC is defined as breast cancer negative for all three markers, and a significant overlap of biological and clinical characteristics between TNBC and basal-like breast cancers, resulting in more than 85% of TNBC belong to basal-like breast cancer [7]. These molecular differences are associated with histopathological and clinical differences as well as variations in treatment response and prognosis, implicating possible differences in metabolic features. Previous studies indicate elevated expression of the glycolysis-related proteins GLUT-1 and CAIX in basal-like type/TNBC breast cancers [8,9] and elevated expression of the glutaminolysis-related protein in HER-2 type breast cancers [10], supporting a plausible relation between metabolism and molecular subtype. However, the association between breast cancer subtype and sarcosine metabolism-related protein expression has not been examined. While most research on sarcosine up to date has been done in prostate cancer, there exists considerable similarity and connection between prostate cancer and breast cancer. Firstly, epidemiologic studies show that family history of breast cancer significantly influences the risk for prostate cancer [11,12]. Secondly, genetic studies showed that androgen receptor(AR) alteration, which is important in the development of prostate cancer, is also present in breast cancer [13]. Moreover, studies have shown that the mutations significant for hereditary breast cancer, BRCA1 and BRCA2 mutations, is related to higher risk for prostate cancer [14,15]. Lastly, in a biochemical aspect, the prostate cancer-sensitive marker PSA was also detected in breast cancer [16], and its relation to good prognosis of prostate cancer was reported [17]. Conclusively, the epidemiologic, genetic and biochemical similarities suggest a resemblance in sarcosine metabolism between prostate cancer and breast cancer.

Therefore, in this study, we determined the expression of sarcosine metabolism-related protein in various breast cancer subtypes and investigated the implications of differences in expression pattern according to subtype.

## Materials and methods

### Patient selection

Subjects were selected from among patients diagnosed with invasive breast cancer who received surgical treatment from January 2000 to December 2006 at Severance Hospital. Patients who received pre-operative hormone or chemotherapy were excluded. This study was approved by the Institutional Review Board of Yonsei University Severance Hospital. IRB exempted the informed consent from patients. All cases were reviewed retrospectively by a breast pathologist (Koo JS) using Hematoxylin & Eosin (H&E)-stained slides. Histological grade was assessed using the Nottingham grading system [18]. Clinicopathologic parameters evaluated in each case included patient age at initial diagnosis, lymph node metastasis, tumor recurrence, distant metastasis, and patient survival.

### Tissue microarray

A representative area was selected on an H&E-stained slide, and a corresponding spot was marked on the surface of the paraffin block. Using a biopsy needle, the selected area was punched out, and a 3-mm tissue core was transferred to a 6 × 5 recipient block. Two tissue cores of invasive tumor were extracted to minimize extraction bias. Each tissue core was assigned a unique tissue microarray location number that was linked to a database containing other clinicopathologic data.

### Immunohistochemistry

Antibodies used for immunohistochemistry are listed in Additional file 1: Table S1. All immunohistochemistry was performed with formalin-fixed, paraffin-embedded tissue sections. Briefly, 5-μm-thick sections were obtained with a microtome, transferred onto adhesive slides, and dried at 62°C for 30 minutes. After incubation with primary antibodies, immunodetection was performed with biotinylated anti-mouse immunoglobulin, followed by peroxidase-labeled streptavidin using a labeled streptavidin biotin kit with 3,3′-diaminobenzidine chromogen as the substrate. The primary antibody incubation step was omitted in the negative control. Positive control tissue was used as per the manufacturer’s recommendation. Slides were counterstained with Harris hematoxylin.

### Interpretation of immunohistochemical staining

All immunohistochemical markers were accessed by light microscopy. A cut-off value of 1% or more positively stained nuclei was used to define ER and PR positivity [19]. HER-2 staining was analyzed according to the American Society of Clinical Oncology (ASCO)/College of American Pathologists (CAP) guidelines using the following categories: 0 = no immunostaining; 1+ = weak incomplete membranous staining, less than 10% of tumor cells; 2+ = complete membranous staining, either uniform or weak in at least 10% of tumor cells; and 3+ = uniform intense membranous staining in at least 30% of tumor cells [20]. HER-2 immunostaining was considered positive when strong (3+) membranous staining was observed, whereas cases with 0 to 1+ were regarded as negative. Cases showing 2+ HER-2 expression were evaluated for HER-2 amplification by fluorescent *in situ* hybridization (FISH).

Immunohistochemical markers for GNMT, SARDH, and PIPOX were accessed by light microscopy. Immunohistochemical staining was calculated as the product of the proportion of stained cells and immunostaining intensity. Proportion of stained cells was stratified as 0: negative, 1: less than 30% positive, and 2: equal to or more than 30% positive, while immunostaining intensity was stratified as 0: negative, 1: weak, 2: moderate, and 3: strong. Immunohistochemistry was deemed negative when the product of the proportion of stained cells and immunostaining intensity was 0–1 and positive when the product was 2–6 [21]. Ki-67 labeling index (LI) was defined as the percentage of cancer cells with a Ki-67-positive nucleus.

### FISH analysis

Before FISH analysis, invasive tumors were examined on hematoxylin-eosin stained slides. FISH was subsequently performed on the tested tumor using a PathVysion *HER-2* DNA Probe Kit (Vysis, Downers Grove, IL, USA) in accordance with the manufacturer’s instructions. HER-2 gene copy number was evaluated using an epifluorescence microscope (Olympus, Tokyo, Japan). At least 60 tumor cell nuclei in three separate regions were investigated for *HER-2* and chromosome 17 signals. *HER-2* gene amplification was determined according to the ASCO/CAP guidelines [20]. An absolute *HER-2* gene copy number less than 4 or a *HER-2* gene/chromosome 17 copy number ratio (HER-2/Chr17 ratio) less than 1.8 was considered *HER-2* negative. An absolute *HER-2* copy number between 4 and 6 or a HER-2/Chr17 ratio between 1.8 and 2.2 was considered *HER-2* equivocal. An absolute *HER-2* copy number greater than 6 or a HER-2/Chr17 ratio higher than 2.2 was considered *HER-2* positive.

### Tumor phenotype classification

In this study, we classified breast cancer phenotypes according to the immunohistochemistry results for ER, PR, HER-2, Ki-67 and FISH results for HER-2 as follows [22]: *luminal A type*, ER or/and PR positive, HER-2 negative and Ki-67 LI <14%; *Luminal B type*, (HER-2 negative) ER or/and PR positive, HER-2 negative and Ki-67 LI ≥14%; (HER-2 positive) ER or/and PR positive and HER-2 overexpressed or/and amplified; *HER-2 overexpression type*, ER and PR negative and HER-2 overexpressed or/and amplified; *TNBC type*: ER, PR, and HER-2 negative.

### Sarcosine metabolism phenotype

Sarcosine metabolism phenotype was classified according to immunohistochemistry results for GNMT, SARDH, and PIPOX. High sarcosine type was defined as GNMT(+)/SARDH and PIPOX(-), low sarcosine type was defined as GNMT(-)/SARDH or PIPOX(+), intermediate sarcosine type was defined as GNMT(+)/SARDH or PIPOX(+), and null type was defined as GNMT(-)/SARDH and PIPOX(-).

### Statistical analysis

Data were analyzed using SPSS for Windows, Version 12.0 (SPSS Inc., Chicago, IL, USA). For determination of statistical significance, Student’s *t* and Fisher’s exact tests were used for continuous and categorical variables, respectively. In the case of analyzing data with multiple comparisons, a corrected p-value with the application of the Bonferroni multiple comparison procedure was used. Statistical significance was set to p < 0.05. Kaplan-Meier survival curves and log-rank statistics were employed to evaluate time to tumor recurrence and overall survival. Multivariate regression analysis was performed using the Cox proportional hazards model.

## Results

### Patient clinicopathologic characteristics

Of a total of 721 subjects, there were 303 (42.0%) luminal A, 169 (23.4%) luminal B, 71 (9.8%) HER-2 type, and 178(24.7%) TNBC. Clinicopathologic analysis revealed that TNBC tumors had a higher histological grade (p < 0.001), higher T stage (p = 0.002), and higher Ki-67 LI (p < 0.001) than the other tumor sub-types. In contrast, the HER-2 type was associated with older patient age (p = 0.012), higher tumor recurrence rate (p = 0.002), and higher mortality (p = 0.001) than the other subtypes (Table 1).

**Table 1:** Clinicopathologic characteristics of patients according to breast cancer phenotype

**Parameter**	**Total (n =721) (%)**	**Luminal A (n =303) (%)**	**Luminal B (n =169) (%)**	**HER-2 (n =71) (%)**	**TNBC (n =178) (%)**	***P*-value**
Age (years)						**0.012**
≤50	428 (59.4)	173 (57.1)	113 (66.9)	32 (45.1)	110 (61.8)	
>50	293 (40.6)	130 (42.9)	56 (33.1)	39 (54.9)	68 (38.2)	
Histologic grade						**<0.001**
I/II	485 (67.3)	275 (90.8)	112 (66.3)	37 (52.1)	61 (34.3)	
III	236 (32.7)	28 (9.2)	57 (33.7)	34 (47.9)	117 (65.7)	
Tumor stage						**0.002**
T1	355 (49.2)	169 (55.8)	86 (50.9)	32 (45.1)	68 (38.2)	
T2/T3	366 (50.8)	134 (44.2)	83 (49.1)	39 (54.9)	110 (61.8)	
Nodal metastasis						0.162
Absent	427 (59.2)	173 (57.1)	93 (55.0)	44 (62.0)	117 (65.7)	
Present	294 (40.8)	130 (42.9)	76 (45.0)	27 (38.0)	61 (34.3)	
Estrogen receptor status						**<0.001**
Negative	259 (35.9)	5 (1.7)	5 (3.0)	71 (100.0)	178 (100.0)	
Positive	462 (64.1)	298 (98.3)	164 (97.0)	0 (0.0)	0 (0.0)	
Progesterone receptor status						**<0.001**
Negative	347 (48.1)	50 (16.5)	48 (28.4)	71 (100.0)	178 (100.0)	
Positive	374 (51.9)	253 (83.5)	121 (71.6)	0 (0.0)	0 (0.0)	
HER-2 status						**<0.001**
Negative	565 (78.4)	303 (100.0)	84 (49.5)	0 (0.0)	178 (100.0)	
Positive	156 (21.6)	0 (0.0)	85 (50.3)	71 (100.0)	0 (0.0)	
Ki-67 LI (%)						**<0.001**
≤14	409 (56.7)	303 (100.0)	49 (29.0)	29 (40.8)	28 (15.7)	
>14	312 (43.3)	0 (0.0)	120 (71.0)	42 (59.2)	150 (84.3)	
Tumor recurrence	63 (8.7)	15 (5.0)	13 (7.7)	11 (15.5)	24 (13.5)	**0.002**
No. of patient deaths	61 (8.5)	13 (4.3)	13 (7.7)	11 (15.5)	24 (13.5)	**0.001**
Duration of clinical follow-up (months, mean ± SD)	69.9 ± 31.2	71.9 ± 29.0	70.0 ± 30.1	66.1 ± 34.9	68.1 ± 34.1	0.419

Bold number represents p<0.05.

TNBC, triple negative breast cancer.

### Expression of sarcosine metabolism-related proteins according to tumor phenotype

**Figure 1:**
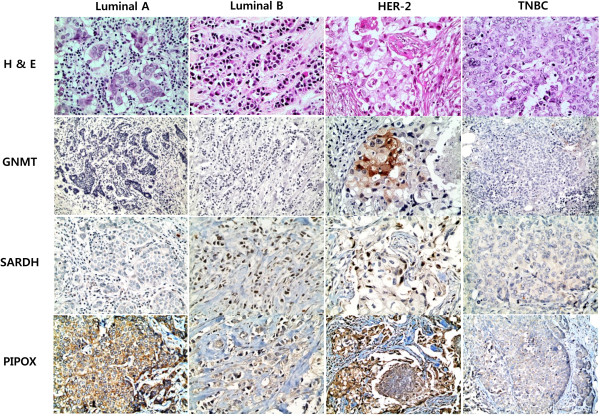
Expression of sarcosine metabolism-related proteins according to the molecular subtype of breast cancer.

**Figure 2:**
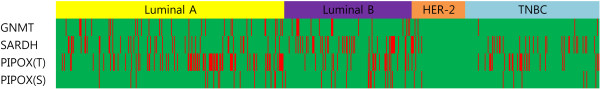
**A heatmap of the expression of sarcosine metabolism-related proteins according to the molecular subtype of breast cancer.** T, tumor, S, stroma, red: positive, green: negative.

Analysis of sarcosine metabolism-related protein expression revealed differences in expression depending on molecular subtype. HER-2 type tumors the most frequently showed the expression of GNMT (p = 0.005), SARDH (p = 0.012), tumoral PIPOX (p = 0.008), and stromal PIPOX (p < 0.001), while TNBC tumors the least frequently exhibited the expression of all four proteins (Table 2, Figure 1, and Figure 2). Sarcosine metabolism phenotypes also varied according to molecular subtype; HER-2 type tumors had the highest ratio of high sarcosine type, while TNBC tumors had the highest ratio of null type (p = 0.003, Table 3). Clinically, there were statistically significant differences in ER expression (p = 0.049), PR expression (p = 0.011), Ki-67 LI (p = 0.007), and tumor recurrence (p = 0.022) according to sarcosine metabolism type. Intermediate sarcosine type had the highest rate of ER and PR positivity with a low Ki-67 LI, while null type tumors had the lowest ER and PR positivity with a high Ki-67 LI. High sarcosine type showed the highest tumor recurrence rate (Table 4).

**Table 2:** Expression of metabolism-related proteins according to breast cancer subtype

**Parameter**	**Total (n =721) (%)**	**Luminal A (n =303) (%)**	**Luminal B (n =169) (%)**	**HER-2 (n =71) (%)**	**TNBC (n =178) (%)**	***P*-value**
GNMT						**0.005**
Negative	664 (92.1)	273 (90.1)	153 (90.5)	63 (88.7)	175 (98.3)	
Positive	57 (7.9)	30 (9.9)	16 (9.5)	8 (11.3)	3 (1.7)	
SARDH						**0.012**
Negative	597 (82.8)	261 (86.1)	130 (76.9)	53 (74.6)	153 (86.0)	
Positive	124 (17.2)	42 (13.9)	39 (23.1)	18 (25.4)	25 (14.0)	
PIPOX (T)						**0.008**
Negative	570 (79.1)	225 (74.3)	143 (84.6)	52 (73.2)	150 (84.3)	
Positive	151 (20.9)	78 (25.7)	26 (15.4)	19 (26.8)	28 (15.7)	
PIPOX (S)						**<0.001**
Negative	671 (93.1)	287 (94.7)	154 (91.1)	58 (81.7)	172 (96.6)	
Positive	50 (6.9)	16 (5.3)	15 (8.9)	13 (18.3)	6 (3.4)	

Bold number represents p<0.05.

TNBC, triple negative breast cancer.

**Table 3:** Sarcosine metabolism phenotypes of different breast cancer subtypes

**Parameter**	**Total (N =721) (%)**	**Luminal A (n =303) (%)**	**Luminal B (n =169) (%)**	**HER-2 (n =71) (%)**	**TNBC (n =178) (%)**	***P*-value**
*Sarcosine metabolic type*						**0.003**
High sarcosine type	36 (5.0)	16 (5.3)	12 (7.1)	7 (9.9)	1 (0.6)	
Intermediate sarcosine type	21 (2.9)	14 (4.6)	4 (2.4)	1 (1.4)	2 (1.1)	
Low sarcosine type	219 (30.4)	93 (30.7)	54 (32.0)	26 (36.6)	46 (25.8)	
Null type	445 (61.7)	180 (59.4)	99 (58.6)	37 (52.1)	129 (72.5)	

Bold number represents p<0.05.

TNBC, triple negative breast cancer.

**Table 4:** Clinicopathologic characteristics of patients according to sarcosine metabolism type

**Parameter**	**Total N =721 (%)**	**High sarcosine type n = 36 (%)**	**Intermediate sarcosine type n = 21 (%)**	**Low sarcosine type n = 219 (%)**	**Null type n = 445 (%)**	***P*-value**
Age (years)						0.200
≤50	428 (59.4)	26 (72.2)	13 (61.9)	120 (54.8)	269 (60.4)	
>50	293 (40.6)	10 (27.8)	8 (38.1)	99 (45.2)	176 (39.6)	
Histologic grade						0.351
I/II	485 (67.3)	29 (80.6)	13 (61.9)	147 (67.1)	296 (66.5)	
III	236 (32.7)	7 (19.4)	8 (38.1)	72 (32.9)	149 (33.5)	
Tumor stage						0.082
T1	355 (49.2)	12 (33.3)	10 (47.6)	120 (54.8)	213 (47.9)	
T2/T3	366 (50.8)	24 (66.7)	11 (52.4)	99 (45.2)	232 (52.1)	
Nodal metastasis						0.799
Absent	427 (59.2)	22 (61.1)	14 (66.7)	125 (57.1)	266 (59.8)	
Present	294 (40.8)	14 (38.9)	7 (33.3)	94 (42.9)	179 (40.2)	
Estrogen receptor status						**0.049**
Negative	259 (35.9)	9 (25.0)	3 (14.3)	75 (34.2)	172 (38.7)	
Positive	462 (64.1)	27 (75.0)	18 (85.7)	144 (65.8)	273 (61.3)	
Progesterone receptor status						**0.011**
Negative	347 (48.1)	13 (36.1)	4 (19.0)	102 (46.6)	228 (51.2)	
Positive	374 (51.9)	23 (63.9)	17 (81.0)	117 (53.4)	217 (48.8)	
HER-2 status						0.072
Negative	565 (78.4)	24 (66.7)	17 (81.0)	163 (74.4)	361 (81.1)	
Positive	156 (21.6)	12 (33.3)	4 (19.0)	56 (25.6)	84 (18.9)	
Ki-67 LI (%)						**0.007**
≤14	409 (56.7)	21 (58.3)	17 (81.0)	138 (63.0)	233 (52.4)	
>14	312 (43.3)	15 (41.7)	4 (19.0)	81 (37.0)	212 (47.6)	
Tumor recurrence	63 (8.7)	8 (22.2)	1 (4.8)	15 (6.8)	39 (8.8)	**0.022**
No. of patient deaths	61 (8.5)	5 (13.9)	1 (4.8)	20 (9.1)	35 (7.9)	0.558

Bold number represents p<0.05.

TNBC, triple negative breast cancer.

### Correlations between expression of sarcosine metabolism-related proteins and clinicopathologic factors

Analysis of sarcosine metabolism-related protein expression and clinicopathologic parameters showed an association between PR positivity and GNMT expression (p = 0.016), between HER-2 positivity and SARDH expression (p = 0.016) and stromal PIPOX expression (p = 0.004). Moreover, tumoral PIPOX expression was associated with lower Ki-67 LI (p < 0.001, Table 5).

**Table 5:** Correlations between the expression of sarcosine metabolism-related proteins and clinicopathologic parameters

**Parameter**	**GNMT**			**SARDH**			**PIPOX in tumor**			**PIPOX in stroma**		
	**Negative n =664 (%)**	**Positive n = 57 (%)**	*P*-value*	**Negative n =597 (%)**	**Positive n = 124 (%)**	*P*-value*	**Negative n = 570 (%)**	**Positive n = 151 (%)**	*P*-value*	**Negative n = 671 (%)**	**Positive n =50 (%)**	*P*-value*
Age (years)			0.588			0.336			0.620			1.088
≤50	389 (58.6)	39 (68.4)		363 (60.8)	65 (52.4)		346 (60.7)	82 (54.3)		402 (59.9)	26 (52.0)	
>50	275 (41.4)	18 (31.6)		234 (39.2)	59 (47.6)		224 (39.3)	69 (45.7)		269 (40.1)	24 (48.0)	
Histologic grade			1.128			3.068			3.036			1.024
I/II	443 (66.7)	42 (73.7)		403 (67.5)	82 (66.1)		385 (67.5)	100 (66.2)		455 (67.8)	30 (60.0)	
III	221 (33.3)	15 (26.3)		194 (32.5)	42 (33.9)		185 (32.5)	51 (33.8)		216 (32.2)	20 (40.0)	
Tumor stage			0.376			1.316			0.308			3.644
T1	333 (50.2)	22 (38.6)		289 (48.4)	66 (53.2)		271 (47.5)	84 (55.6)		330 (49.2)	25 (50.0)	
T2/T3	331 (49.8)	35 (61.4)		308 (51.6)	58 (46.8)		299 (52.5)	67 (44.4)		341 (50.8)	25 (50.0)	
Nodal metastasis			2.116			3.640			2.604			2.524
Absent	391 (58.9)	36 (63.2)		353 (59.1)	74 (59.7)		340 (59.6)	87 (57.6)		399 (59.5)	28 (56.0)	
Present	273 (41.1)	21 (36.8)		244 (40.9)	50 (40.3)		230 (40.4)	64 (42.4)		272 (40.5)	22 (44.0)	
Estrogen receptor status			0.060			3.644			1.672			2.132
Negative	247 (37.2)	12 (21.1)		215 (36.0)	44 (35.5)		209 (36.7)	50 (33.1)		239 (35.6)	20 (40.0)	
Positive	417 (62.8)	45 (78.9)		382 (64.0)	80 (64.5)		361 (63.3)	101 (66.9)		432 (64.4)	30 (60.0)	
Progesterone eceptor status			**0.004**			1.572			0.204			3.020
Negative	330 (49.7)	17 (29.8)		283 (47.4)	64 (51.6)		285 (50.0)	62 (41.1)		324 (48.3)	23 (46.0)	
Positive	334 (50.3)	40 (70.2)		314 (52.6)	60 (48.4)		285 (50.0)	89 (58.9)		347 (51.7)	27 (54.0)	
HER-2 status			0.876			**0.004**			3.072			**0.001**
Negative	524 (78.9)	41 (71.9)		480 (80.4)	85 (68.5)		448 (78.6)	117 (77.5)		535 (79.7)	30 (60.0)	
Positive	140 (21.1)	16 (28.1)		117 (19.6)	39 (31.5)		122 (21.4)	34 (22.5)		136 (20.3)	20 (40.0)	
Ki-67 LI (%)			0.456			3.156			**<0.001**			1.280
≤14	371 (55.9)	38 (66.7)		340 (57.0)	69 (55.6)		303 (53.2)	106 (70.2)		384 (57.2)	25 (50.0)	
>14	293 (44.1)	19 (33.3)		257 (43.0)	55 (44.4)		267 (46.8)	45 (29.8)		287 (42.8)	25 (50.0)	

Bold number represents p<0.05.

*p-value was calculated by Bonferroni method.

### Impact of expression of sarcosine metabolism-related proteins on patient prognosis

**Figure 3:**
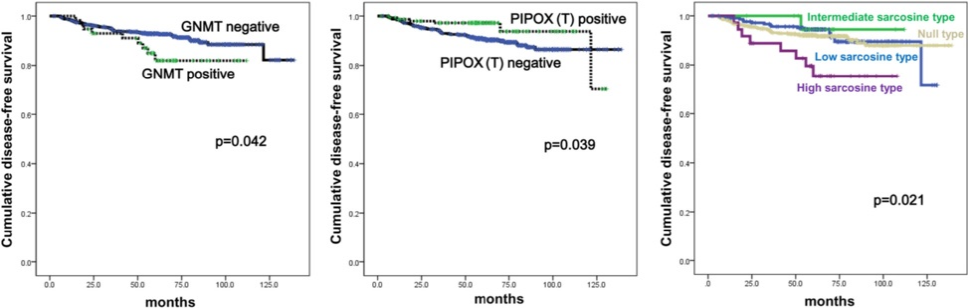
Disease-free survival and overall survival according to the expression of sarcosine metabolism-related proteins and sarcosine metabolism phenotype.

To investigate the potential effects of sarcosine metabolism-related protein expression on prognosis, univariate analysis was performed on all cases regardless of subtype. Factors associated with shorter disease-free survival (DFS) were GNMT expression (p = 0.042), tumoral PIPOX negativity (p = 0.039), and high sarcosine type (p = 0.021, Table 6 and Figure 3). Multivariate Cox analysis revealed that higher T stage (hazard ratio: 2.123, 95% CI: 1.167-3.861, p = 0.014), lymph node metastasis (hazard ratio: 2.344, 95% CI: 1.389-3.956, p = 0.001), and GNMT expression (hazard ratio: 2.408, 95% CI: 1.154-5.024, p = 0.019) were independent factors associated with shorter DFS. Additionally, higher T stage (hazard ratio: 1.829, 95% CI: 1.028-3.255, p = 0.040) and lymph node metastasis (hazard ratio: 1.971, 95% CI: 1.166-3.333, p = 0.011) were independent factors associated with shorter overall survival (OS) (Table 7).

**Table 6:** Univariate analysis of the impact of expression of serine/glycine metabolism-related proteins in breast cancers on disease-free survival and overall survival by the log-rank test

**Parameter**	**Number of patients/recurrence/death**	**Disease-free survival**		**Overall survival**	
		**Mean survival (95% CI) months**	***P *-value**	**Mean survival (95% CI) months**	***P *-value**
GNMT			**0.042**		0.498
Negative	664/54/55	127 (123–130)		129 (127–132)	
Positive	57/9/6	98 (91–106)		126 (116–135)	
SARDH			0.767		0.264
Negative	597/53/47	126 (122–130)		130 (127–133)	
Positive	124/10/14	121 (115–127)		124 (117–132)	
PIPOX (T)			**0.039**		0.361
Negative	570/57/52	126 (123–129)		129 (126–131)	
Positive	151/6/9	123 (117–129)		131 (126–135)	
PIPOX (S)			0.927		0.636
Negative	671/59/58	126 (122–130)		129 (126–132)	
Positive	50/4/3	122 (114–130)		126 (119–132)	
*Sarcosine metabolic type*			**0.021**		0.503
High sarcosine type	36/8/5	90 (79–101)		118 (106–130)	
Intermediate sarcosine type	21/1/1	108 (102–114)		132 (122–143)	
Low sarcosine type	219/15/20	120 (115–125)		126 (121–132)	
Null type	445/39/35	127 (124–131)		130 (127–133)	

Bold number represents p<0.05.

**Table 7:** Multivariate analysis of breast-cancer survival

**Included parameters**	**Disease-free survival**			**Overall survival**		
	**Hazard ratio**	**95% CI**	***P*-value**	**Hazard ratio**	**95% CI**	***P*-value**
T stage			**0.014**			**0.040**
T1 versus T2-3	2.123	1.167-3.861		1.829	1.028-3.255	
N stage			**0.001**			**0.011**
N0 versus N1-3	2.344	1.389-3.956		1.971	1.166-3.333	
Histologic grade			0.632			0.545
I/II versus III	1.143	0.661-1.977		0.839	0.475-1.482	
ER status			0.111			0.079
Negative versus Positive	1.795	0.874-3.686		1.888	0.929-3.836	
PR status			0.198			0.070
Negative versus Positive	1.638	0.773-3.473		2.036	0.944-4.393	
HER-2 status			0.469			0.508
Negative versus Positive	1.235	0.697-2.188		1.214	0.683-2.159	
GNMT			**0.019**			0.272
Negative versus Positive	2.408	1.154-5.024		1.622	0.684-3.849	
PIPOX (T)			0.069			0.563
Negative versus Positive	0.457	0.196-1.064		0.810	0.397-1.652	

Bold number represents p<0.05.

**Figure 4:**
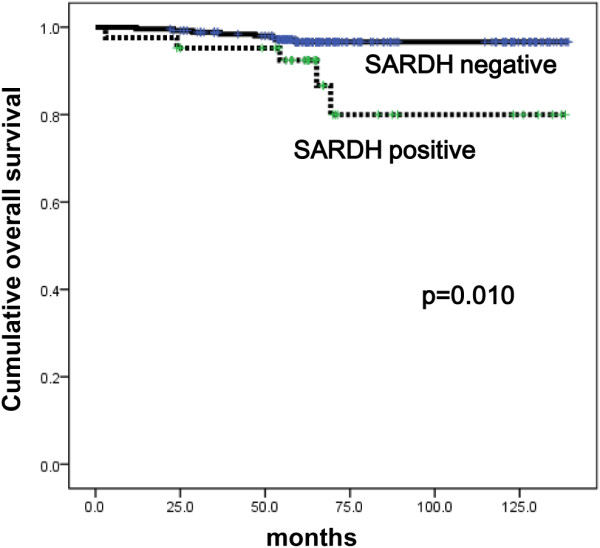
Overall survival according to the expression of SARDH in luminal A type breast cancer.

**Figure 5:**
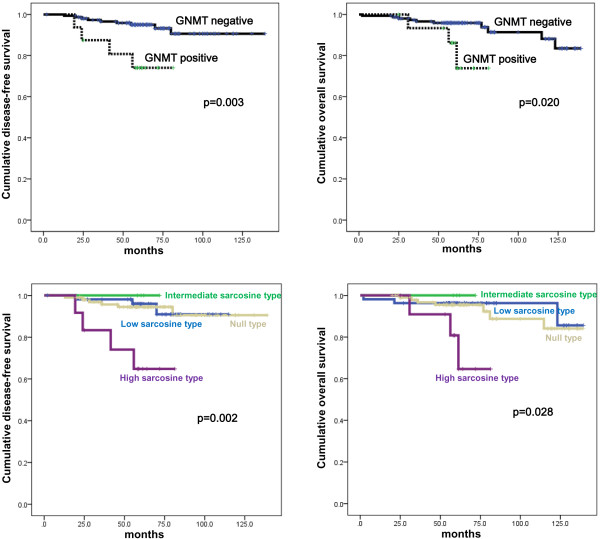
Disease-free survival and overall survival according to the expression of GNMT and sarcosine metabolism phenotype in luminal B type breast cancer.

The effect of sarcosine metabolism-related protein expression on prognosis according to molecular subtype was studied. In luminal A, univariate analysis showed that SARDH expression was associated with shorter OS (p = 0.010, Figure 4), and SARDH expression was an independent factor for shorter OS (hazard ratio: 3.793, 95% CI: 1.231-11.68, p = 0.020). In luminal B, GNMT expression (p = 0.003 and 0.020, respectively) and high sarcosine type (p = 0.002 and 0.028, respectively) were correlated with both shorter DFS and shorter OS (Figure 5), but no correlation was found in multivariate Cox analysis (Additional file 1: Table S2 and Additional file 1: Table S3).

## Discussion

We investigated the expression of sarcosine metabolism-related proteins in breast cancer, with a focus on molecular subtypes of breast cancer. GNMT, SARDH, and PIPOX showed the highest expression levels in HER-2 type cancer. Sarcosine has mostly been researched in prostate cancer; studies have shown that increased sarcosine level is associated with cancer progression, and *in vitro* and *in vivo* models have suggested a correlation between prostate cancer growth and progression [2]. Key results include the induction of invasive phenotypes by injection of sarcosine into benign prostate cells [1] and increased sarcosine levels in prostate cancer tissue [23]. Increased sarcosine levels in prostate cancer can be useful in cancer detection. Reports revealed that sarcosine had higher predictive value in detecting prostate cancer in tissue biopsy than PSA, particularly when PSA level was between 2 to 10 ng/ml or the gray zone [1], or when it was less than 4 ng/ml [24,25]. Moreover, sarcosine levels and GNMT expression were also increased in tissue and feces of experimental azoxymethane-induced colorectal cancer [26].

Unfortunately, there is no prior research on sarcosine in breast cancers against which to compare our study findings. Reports indicate strong correlations between sarcosine metabolism-related proteins and prostate cancer. Compared to normal prostate tissue, prostate cancer tissue showed higher expression of GNMT, the sarcosine generating enzyme, and lower expression of SARDH and PIPOX, which are sarcosine metabolizing enzymes, suggesting a correlation between sarcosine level and the expression of sarcosine metabolism-related proteins [2]. Although we did not measure sarcosine levels directly from breast cancer tissues, we used expression levels of the major sarcosine metabolism-related proteins GNMT, SARDH, and PIPOX as surrogate for sarcosine level. The expression rate of GNMT, SARDH, and PIPOX were higher in HER-2 type relative to the others subtypes. Moreover, the proportion of high sarcosine type [GNMT(+)/SARDH and PIPOX(-)] was highest in HER-2 type tumors, suggesting that sarcosine levels are higher in HER-2 type breast cancers than in other molecular subtypes. The mechanism underlying higher expression of sarcosine metabolism-related protein in HER-2 type tumors requires elucidation in further validation studies. One hypothesis is that sarcosine, HER-2, and the androgen receptor influence each other. A previous study showed that sarcosine increased both HER-2 mRNA and protein levels in an androgen-dependent prostate cancer cell line, suggesting an association among the androgen receptor, HER-2, and sarcosine [27]. Correspondingly, the molecular apocrine type in breast cancer defined as ER-negative and AR-positive breast cancer showed significant overlap with the HER-2-enriched group on gene profiling analysis [28]. Approximately 50% of molecular apocrine type exhibited HER-2 amplification/overexpression, strongly implying a correlation between AR and HER-2. Further research on the effects of sarcosine on HER-2 according to AR status will help elucidate the mechanism of association among these key molecules in breast cancer. Another interesting finding is the correlation between GNMT positivity/PIPOX negativity and shorter DFS. A previous prostate cancer study revealed that increased levels of sarcosine induce invasion and intravasation [2], supporting the role of sarcosine as an oncometabolite in prostate cancer. A separate study showed that serum sarcosine levels were significantly higher in patients with metastatic prostate cancer [29]. It follows that sarcosine levels would be higher in breast cancer with positive GNMT and negative PIPOX, which could be the reason for shorter DFS in such breast cancer. Moreover, analysis of sarcosine metabolism phenotype revealed that patients with sarcosine-high type [GNMT(+)/SARDH and PIPOX(-)] tumors had a shorter DFS than patients with other breast cancer tumor types, suggesting that sarcosine level is a prognostic factor in breast cancer. A previous study reported a potential association between high GNMT cytoplasmic expression in prostate cancer and lower DFS rate [30], consistent with our findings. In contrast, research on hepatic cholangiocarcinoma suggests that GNMT expression was a favorable prognostic marker [31]. It is highly likely that sarcosine may have different roles according to the type and subtype of cancer. Further validation studies to investigate the correlation between sarcosine level and cancer prognosis are necessary. That sarcosine metabolism-related proteins expression was significantly lower in TNBC-type tumors relative to the other subtypes was an unexpected result. TNBC tumors have highly aggressive histological characteristics such as high histologic grade, high levels of mitosis and tumor necrosis [7]. Accordingly, we expected TNBC tumor specimens to exhibit strong metabolic activity, as previous studies have reported increased expression of Glut-1 and CAIX in TNBC compared to other breast cancer subtypes [8,9]. Our results imply that sarcosine does not contribute significantly to the aggressiveness of TNBC. This topic requires further investigation.

## Conclusion

The expressions of sarcosine metabolism-related proteins varied according to subtype of breast cancer; expression of these proteins was elevated in HER-2 type and decreased in TNBC. We also demonstrated that sarcosine metabolism-related proteins had prognostic utility in breast cancer patients.

## Abbreviations

SAM: *S*-adenosylmethionine; GNMT: Glycine *N*-methyltransferase; SARDH: Sarcosine-metabolizing enzymes - sarcosine dehydrogenase; PIPOX: I-pipecloioc acid oxidase; ER: Estrogen receptor; PR: Progesterone receptor; TNBC: Triple negative breast cancer; CAIX: Carbonic anhydrase; H&E: Hematoxylin & Eosin; ASCO: American society of clinical oncology; CAP: College of American Pathologists; DFS: Disease-free survival; OS: Overall survival.

## Competing interests

The authors declare that they have no competing interests.

## Authors’ contributions

JKY participated in the design of the study and performed the statistical analysis and drafted the manuscript. DHK carried out the immunochemistry. JSK conceived the study, and participated in its design and coordination and helped to draft the manuscript. All authors read and approved the final manuscript.

## Supplementary Material

Additional file 1: Table S1 Source, clone, and dilutions of antibodies used in this study. **Table S2.** Multivariate analysis of breast-cancer survival in patients with luminal B type cancer. **Table S3.** Multivariate analysis of breast-cancer survival in patients with luminal A type cancer. Click here for file
