# Supplementary material for: Implications of differences in expression of sarcosine metabolism-related proteins according to the molecular subtype of breast cancer
Source: J Transl Med. 2014 May 28;12:149. doi: 10.1186/1479-5876-12-149 (PMC4045904; doi:10.1186/1479-5876-12-149)
Supplement: Additional file 1: Table S1 — Source, clone, and dilutions of antibodies used in this study. Table S2. Multivariate analysis of breast-cancer survival in patients with luminal B type cancer. Table S3. Multivariate analysis of breast-cancer survival in patients with luminal A type cancer. [file 1479-5876-12-149-S1.doc]

| **Supplementary Table 1.** Source, clone, and dilutions of antibodies used in this study. | | | |
| --- | --- | --- | --- |
| **Antibody** | **Company** | **Clone** | **Dilution** |
| *Sarcosine metabolism-related* |  |  |  |
| GNMT | Abcam, Cambridge, UK | Polyclonal | 1:100 |
| SARDH | Abcam, Cambridge, UK | Polyclonal | 1:100 |
| PIPOX | Abcam, Cambridge, UK | Polyclonal | 1:100 |
| *Molecular subtype-related* |  |  |  |
| ER | Thermo Scientific, San Diego, CA, USA | SP1 | 1:100 |
| PR | DAKO, Glostrup, Denmark | PgR | 1:50 |
| HER-2 | DAKO, Glostrup, Denmark | Polyclonal | 1:1500 |
| Ki-67 | Abcam, Cambridge, UK | MIB | 1:1000 |

glycine *N*-methyltransferase (GNMT), sarcosine dehydrogenase (SARDH), and l-pipecolic acid oxidase (PIPOX)

| **Supplementary Table 2.** Multivariate analysis of breast-cancer survival in patients with luminal B type cancer. | | | | | | | |
| --- | --- | --- | --- | --- | --- | --- | --- |
| Included parameters | Disease-free survival | | |  | Overall survival | | |
|  | Hazard ratio | 95% CI | *P*-value |  | Hazard ratio | 95% CI | *P*-value |
| T stage |  |  | 0.337 | |  |  | 0.655 |
| T1 versus T2-3 | 2.215 | 0.437-11.224 |  | | 1.382 | 0.337-5.711 |  |
| N stage |  |  | **0.020** | |  |  | **0.029** |
| N0 versus N1-3 | 11.994 | 1.482-97.07 |  | | 5.692 | 1.198-27.04 |  |
| Histologic grade |  |  | 0.914 | |  |  | 0.743 |
| I/II versus III | 1.069 | 0.319-3.576 |  | | 0.817 | 0.245-2.728 |  |
| ER status |  |  | 0.574 | |  |  | 0.352 |
| Negative versus Positive | 1.997 | 0.180-22.19 |  | | 3.107 | 0.286-33.79 |  |
| PR status |  |  | 0.388 | |  |  | 0.427 |
| Negative versus Positive | 1.698 | 0.510-5.659 |  | | 1.626 | 0.490-5.394 |  |
| HER-2 status |  |  | 0.781 | |  |  | 0.974 |
| Negative versus Positive | 1.185 | 0.358-3.925 |  | | 0.981 | 0.306-3.143 |  |
| GNMT |  |  | 0.071 | |  |  | 0.088 |
| Negative versus Positive | 3.675 | 0.895-15.10 |  | | 3.941 | 0.815-19.06 |  |
|  | | | | | | | |
| **Supplementary Table 3**. Multivariate analysis of breast-cancer survival in patients with luminal A type cancer. | | | | | | | |
| Included parameters | Disease-free survival | | |  | Overall survival | | |
|  | Hazard ratio | 95% CI | *P*-value |  | Hazard ratio | 95% CI | *P*-value |
| T stage |  |  | 0.870 | |  |  | 0.482 |
| T1 versus T2-3 | 1.090 | 0.387-3.073 |  | | 0.657 | 0.209=2.063 |  |
| N stage |  |  | 0.724 | |  |  | 0.939 |
| N0 versus N1-3 | 1.203 | 0.431-3.356 |  | | 0.957 | 0.309=2.959 |  |
| Histologic grade |  |  | 0.195 | |  |  | 0.500 |
| I/II versus III | 2.322 | 0.649-8.311 |  | | 1.694 | 0.366-7.838 |  |
| ER status |  |  | N/A | |  |  | N/A |
| Negative versus Positive | N/A | N/A |  | | N/A | N/A |  |
| PR status |  |  | 0.409 | |  |  | 0.205 |
| Negative versus Positive | 1.629 | 0.512-5.188 |  | | 2.154 | 0.657-7.059 |  |
| SARDH |  |  | 0.136 | |  |  | **0.020** |
| Negative versus Positive | 2.408 | 0.758-7.645 |  | | 3.793 | 1.231-11.68 |  |
|  | | | | | | | |
